# Coherent Effects in Charge Transport in Molecular Wires: Toward a Unifying Picture of Long-Range Hole Transfer in DNA

**DOI:** 10.1021/acs.jpclett.0c01996

**Published:** 2020-08-24

**Authors:** Alessandro Landi, Amedeo Capobianco, Andrea Peluso

**Affiliations:** Dipartimento di Chimica e Biologia “A. Zambelli”, Università di Salerno, I-84084 Fisciano, Salerno, Italy

## Abstract

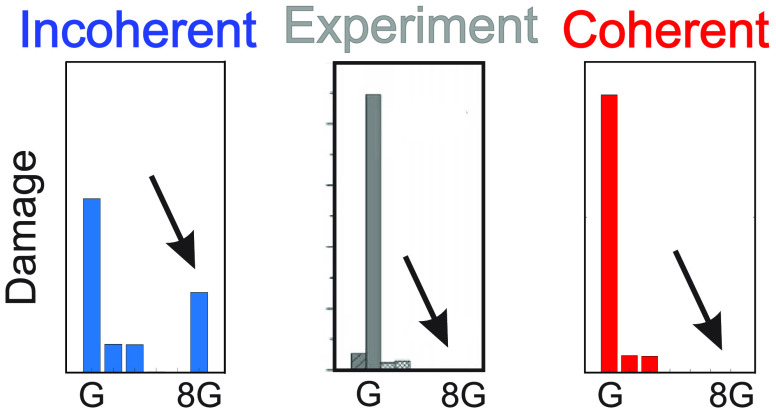

In the framework of a multistep mechanism in which environmental motion triggers comparatively faster elementary electron-transfer steps and stabilizes hole-transfer products, microscopic coherence is crucial for rationalizing the observed yield ratios of oxidative damage to DNA. Interference among probability amplitudes of indistinguishable electron-transfer paths is able to drastically change the final outcome of charge transport, even in DNA oligomers constituted by similar building blocks, and allows for reconciling apparently discordant experimental observations. Properly tailored DNA oligomers appear to be a promising workbench for studying tunneling in the presence of dissipation at the macroscopic level.

Hole transport along the DNA double helix has been extensively studied in the recent decades,^1−8^ both because its possible significance in mutagenesis, carcinogenesis, and aging,^9,10^ and because it has made DNA attractive for applications in molecular electronics,^11−14^ possibly leading to biocompatible and biodegradable devices.^15^

Several pieces of experimental evidence have suggested that charge transport in DNA is characterized by two distinct regimes:^3,16^ a short-range regime, in which hole-transfer (HT) rates exponentially decay with the donor–acceptor (DA) distance, and a long-range regime, where HT rates display a much weaker distance dependence. These results have usually been rationalized in terms of two different mechanisms: one-step coherent hole tunneling (superexchange or flickering resonance) for short DA distances and incoherent multistep hopping for long-range hole transport.^16−21^

Herein, we propose a unifying mechanism of hole transport in DNA, for both short- and long-range regimes, which accounts almost quantitatively for the observed distributions of oxidative damage at DNA nucleobases in different sets of oligomers, and allows for reconciling apparently discordant experimental observations, obtained for two separated sets of DNA oligomers, which, although sharing similar structural motifs, exhibit drastically different charge transport properties.^3,4^

The hole transport mechanism adopted here is a multistep mechanism based on a manifold of fast coherent elementary electron-transfer processes, which take place in resonance conditions, triggered by environmental motions (see Scheme 1). It essentially consists of four steps: (i) an activation step which brings a donor and an acceptor group into vibronic degeneracy; (ii) elementary electron transfer between resonant donor and acceptor groups; (iii) relaxation of non-equilibrium species (including the environment) to their minimum energy structures; (iv) formation of oxidative damage products. In Scheme 1, D^+^(Bridge)A and D(Bridge)A^+^ denote the minimum energy structures with the charge localized on the donor (D) and the acceptor (A) groups; [D^+^(Bridge)A]* and [D(Bridge)A^+^]* indicate the ensembles of structures in which D and A are in vibronic resonance, and *P*_D_ and *P*_A_ denote the products of oxidative damage occurring at the D and A site. *k*_Act_’s are the rate constants of the activation steps, which can be due to either collisions and/or environmental motions. The usual Arrhenius dependence on temperature and activation energy is assumed for *k*_Act_’s:
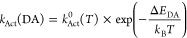
1where Δ*E*_DA_ is the *in situ* electronic hole site energy difference between donor and acceptor and *k*_Act_^0^ (*T*) is the rate constant for bringing into electronic resonance two sites with the same electronic hole site energies. *k*_HT_ is the rate constant of the elementary HT processes, which, because HT occurs only in resonance conditions, applies to both the direct and the reverse steps. In polar solvents, energy relaxation of the activated species [D^+^(Bridge)A]* and [D(Bridge)A^+^]* is mainly provided by solvent response to a nonequilibrium charge distribution; time-resolved spectroscopic measurements have shown that, in water solutions, solvent relaxation occurs on subpicosecond time scales,^22,23^ so that *k*_Rel_ in Scheme 1 has been set to 10^13^ s^–1^ throughout. Finally, *k*_P,D_ and *k*_P,A_ in Scheme 1 denote the rates of formation of the products of oxidative damages.

**Scheme 1:**
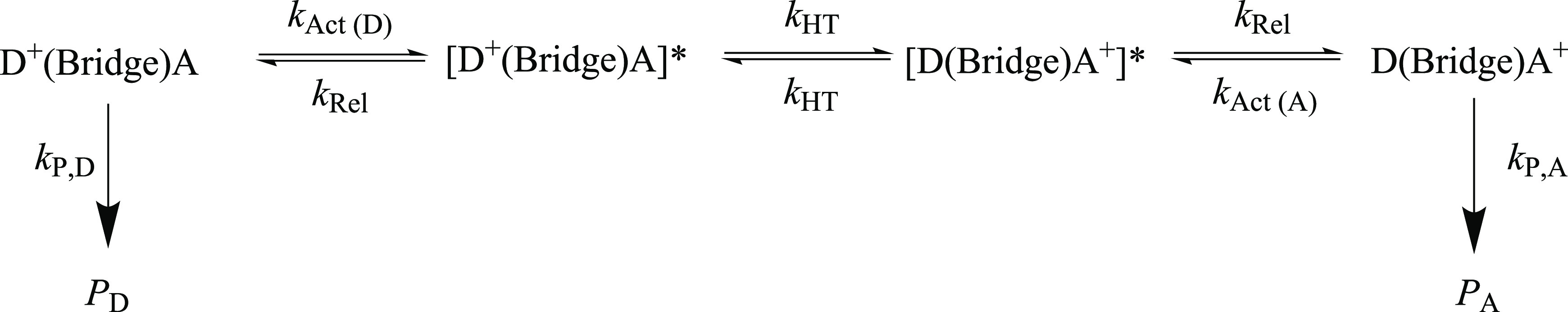


In the framework of the above mechanism, we have considered hole transport in two sets of double-stranded DNA oligomers. The first set (experimentally studied in ref (3)) is constituted by the series of double-stranded ds-G(T)_*n*_GGG (*n* = 1–16) oligomers, (G, guanine; T, thymine), where the single G acts as hole donor, being the site in which the hole is initially photoinjected, and the G triplet acts as hole trap, by virtue of its lower hole site energy.^24,25^ The second set of oligomers (experimentally studied in ref (4)) is constituted by single G’s and G doublets, as low-energy sites for hole transport, differently spaced by either a single thymine or by T multiplets, and by 8-oxo-7,8-dihydroguanine (8-oxoG) as hole trap.^4^ While in the first set of oligomers the amount of oxidative damage at the thermodynamically favored G triplet is always significantly higher than that at the single G, independent of the length of the T bridge, in the second set of oligomers, the T quadruplet behaves as an insurmountable barrier for hole transfer, even in the presence of 8-oxoG, which is a much stronger hole trap than GGG.^4,6^

Among the several oligomers experimentally investigated, we have selected those with *n* = 1–7 of the ds-G(T)_*n*_GGG series and the four double strands shown in Figure 1 which are well representative of all the possible results observed in the second set of oligomers: (i) In the absence of hole traps, the hole spreads over all the low-energy sites (oligomer **1**). (ii) The hole migrates along the strand, splitting over the two end-capping lower energy sites (oligomer **2**). (iii) The hole migrates along the strand, localizing on the deepest trap site (oligomer **3**). (iv) The hole is not able to freely move along the strand and localizes on the less deep GG trap site (oligomer **4**).

**Figure 1:**
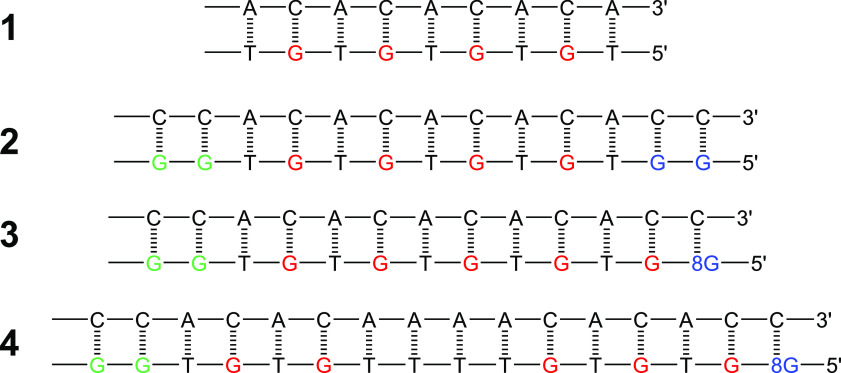
Structures of four DNA oligomers studied in ref (4). A hole is injected on the leftmost G or GG sites, by exciting at 350 nm an anthraquinone group linked at an adenine site of the complementary strand (not shown), producing oxidative damages at G, GG, and 8-oxoG sites; the distribution of oxidative damage yields is revealed by piperidine strand cleavage. Oligomer **1** does not possess trap sites, so that the hole spreads over all the low-energy sites and oxidative damages is observed almost with the same amount at all G sites. Oligomer **2** contains two end-capping trap sites, separated by several TGT steps which act as an efficient hole shuttle: the hole migrates along the strand, splitting to a larger extent over the two end-capping trap sites (>80%) and to a lesser extent onto the single G sites. In oligomer **3**, the distal G doublet is substituted by an 8-oxoG, a deep trap on which most of the oxidative damage (>90%) is observed. In oligomer **4**, a T quadruplet is interposed between the donor GG and the acceptor 8-oxoG; the hole is not able to freely move along the strand and localizes to a larger extent on the G doublet (>90%) and to a lesser extent on single G’s without crossing the T quadruplet bridge.

In order to set the rate constants of elementary hole-transfer processes, we start from the reasonable assumption that the elements of the ensembles of the activated HT species [D(Bridge)A]^+^* differ from each other mainly for solvent configurations, the solute being *grosso modo* in its equilibrium configuration in the majority of the elements of the [D(Bridge)A]^+^* ensemble. Because solvent configuration is irrelevant for the calculation of tunneling rates in resonance conditions, the *k*_HT_’s can be extracted from quantum dynamics simulations of hole transfer for each specific DNA sequence in its minimum-energy configuration.

The adopted model Hamiltonian for hole transfer between nucleobases is based on the usual tight binding approximation and includes as many electronic states as the number of nucleobases.^26−28^ Each electronic state corresponds to a diabatic state in which the electron hole is fully localized on a single nucleobase, with all the others in their neutral form, and is associated with a manifold of vibrational states selected according to their equilibrium position displacements upon hole motion. Accordingly, the adopted hole site energies and intrastrand electronic couplings for stacked nucleobases, reported in Table S1, have been inferred from properly tailored electrochemical and spectroelectrochemical measurements,^25,29^ and their reliability has been extensively tested on several DNA oligomers, providing oxidation potentials in excellent agreement with the available experimental results.^30,31^

Time-dependent *k*_HT_ rate constants have been computed by numerically solving the time-dependent Schrödinger equation (TDSE) with the appropriate initial conditions. In order to keep the kinetic model as simple as possible, time-independent *k*_HT_’s have been obtained by averaging over a half-period of the coherent transition times, i.e., at complete depopulation of the initial state. Indeed, because solvent reorganization is faster than HT dynamics, it is not reasonable to maintain a coherent regime for a long time. The choice of averaging over a half period of coherent oscillation is of course empirical; it has been adopted because solvent reorganization must be triggered by hole dynamics and because it yields, on average, a satisfying agreement with the observed yield ratios for the whole ds-G(T)_*n*_GGG series, with *n* = 1 and 7 (see the Supporting Information).

Hole transfer in ds-G(T)_*n*_GGG oligomers has been the topic of several theoretical works,^16−21^ and a simpler version of our multistep charge transport mechanism (Scheme 1) was recently applied to those oligomers.^27^ Because the charge transport mechanism has been slightly modified for adapting it to more general systems, the new results for the yield ratios of oxidative damage in ds-G(T)_*n*_GGG oligomers are reported in Figure S2 in the Supporting Information together with their experimental counterparts. The results are very similar to the previous ones and have already been extensively discussed in ref (27).

Regarding initial conditions, all quantum dynamics simulations have been carried out at 0 K, and therefore, all of them started from unit population of the ground vibronic state of the G site where charge has been injected. Here, we have made the reasonable approximation that tunneling rates are independent of *T*, but thermal effects can be introduced at affordable computational costs.^32^

The ds-G(T)_*n*_GGG series of oligomers represents a somewhat peculiar case because they are basically two-state systems, with a single donor and a single acceptor species. In contrast, the second set of oligomers contains several sites possessing close hole energies, so that interference among probability amplitudes pertaining to indistinguishable hole paths could arise. Charge transport in the oligomers of this second set can in principle be modeled in two different ways: either as an incoherent sequential hopping, in which transient pairwise resonances promote charge transport, or as a coherent process in which vibronic states of several sites are involved at once. We have taken into consideration both possibilities in our quantum dynamics HT simulations, considering both transient resonances involving single pairs of nucleobases and transient resonances involving all the nucleobases with close hole energies, excluding thymines and cytosines (C) because of their much smaller activation rates.

Hole transfer between G’s in GTG steps of oligomer **1** is fast, of the order of a few tenths of picoseconds (∼0.5) and grows to 5 and 15 ps when the two resonant G’s are separated by off-resonant TGT and TGTGT bridges, respectively (see the Supporting Information, Figure S3). When resonant conditions involve vibronic states of three or more G’s, hole-transfer rates exhibit a much lower distance dependence. The situation is illustrated in Figure 2, where the time evolutions of hole populations in oligomer **1** are reported for two representative cases, one in which only the two ending G’s of **1** are in vibronic resonance, the other in which resonance involves vibronic states of all G’s. While in the first case HT occurs in about 15 ps, in the other case HT is more than 1 order of magnitude faster, exhibiting the peculiarity that the probability of localizing the hole on the ending G’s is significantly higher than over the central G’s.

**Figure 2:**
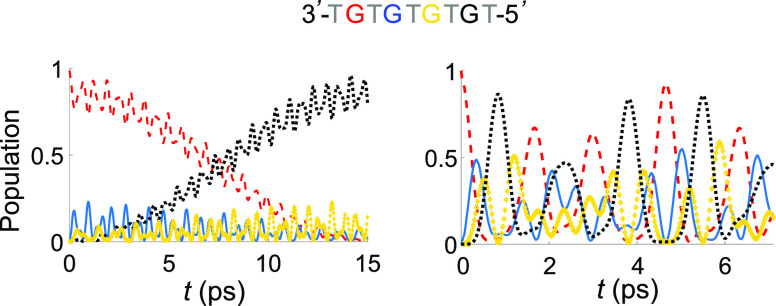
Quantum dynamics population as a function of time for oligomer **1** for two representative cases: resonant G’s separated by an off-resonance TGTGT bridge (left); all four G’s in electronic resonance (right).

Setting the rates of all the possible elementary HT processes from the results of Figures 2 and S3, and setting *k*_Act_^0^ = 2 × 10^10^ s^–1^, the only adjustable parameter in the kinetic model used unaltered throughout, the distribution of oxidative damages in system **1** predicted by our multistep mechanism, generalized to the case of several donor/acceptor species (see the Supporting Information), is shown in Figure 3a, **1** together with experimental results. The rate of formation of oxidative damage *k*_P_ has been set from the rate of deprotonation of G (1 × 10^7^ s^–1^) in double-stranded oligonucleotides, which has been accurately measured by different groups.^33,34^ That choice for *k*_P_ arises because deprotonation is likely the first step of irreversible damage at guanine sites and certainly a process which takes nucleobases out of play for HT. Both the incoherent hopping and the coherent charge transport model predict a roughly equal amount of oxidative damage over all the G nucleobases, but while the former yields a slightly decreasing amount of damage as the HT distance increases, the latter yields larger amounts of damage at the two end-capping nucleobases (see Figure 3a, **1**), in better agreement with experimental observations.^4^

**Figure 3:**
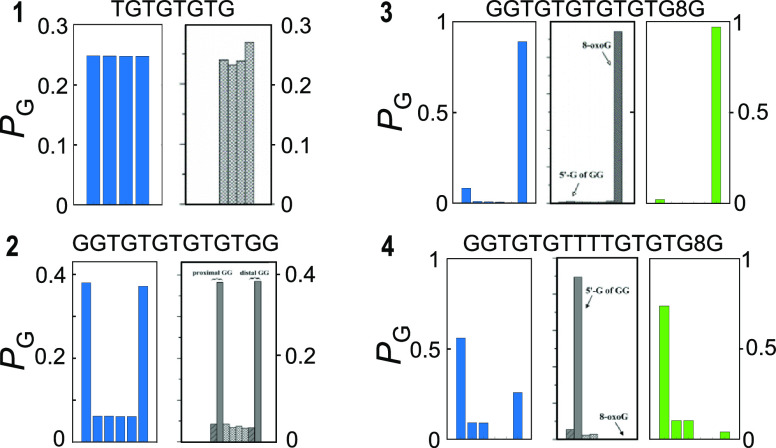
Predicted and observed oxidative damage yields for oligomers **1**–**4** (a−d) obtained by numerically solving the set of ordinary differential equations associated with the kinetic model of Scheme 1, generalized to the case of several donor/acceptor species (see the Supporting Information). Predicted values in blue (on the left in each panel) refer to the incoherent multistep hopping mechanism, with rates extracted from quantum dynamics simulation of hole transfer between pairs of resonant nucleobases. Predicted values in green (on the right in each panel) refer to coherent hole motion occurring when electronic resonance involves more than two nucleobases, with hole-transfer rates extracted from quantum dynamics simulations which include all indistinguishable paths at once. Experimental data, in gray, have been adapted with permission from ref (4). Copyright 2006 American Chemical Society. Note that damage yields for the two G’s constituting a GG step are reported separately in the experimental data, whereas they are summed up in the theoretical data.

Predicted transition times for hole transfer in oligomer **2** are similar to those of oligomer **1** and are reported in the Supporting Information, together with the kinetic schemes and rate constants. As for **1**, *k*_P_’s have been set as the rates of deprotonation of G^•+^ and GG^•+^, 1 × 10^7^ and 3 × 10^6^ s^–1^, respectively.^33,34^ The predicted *P*_GG_/*P*_G_ ratio is ∼10, in line with the experimental outcome (∼42/4.5), both for the incoherent and the coherent mechanism (see Figure 3b, **2**).

Oligomer **3** differs from oligomer **2** by the presence of a 8-oxoG, which exhibits a much lower oxidation potential than GG,^35^ which, according to eq 1, makes 8-oxoG a deep trap which a hole cannot escape from. Because exoergonic HT between adjacent nucleobases is predicted to occur on subpicosecond time scales,^36^ we have assumed that hole localization on 8-oxoG is irreversibly formed upon HT from the adjacent G, occurring with the same time scales on which fast decay of the activated species occurs. With that assumption, leaving unaltered all the other parameters, the predicted yields of oxidative damage at 8-oxoG site are 87% and 93% for the incoherent and coherent model, respectively, the last value being in very good agreement with the observed one (∼95%) (see Figure 3c, **3**).

Oligomer **4** is the most interesting sequence of the second set, because no oxidative damage was experimentally observed at the 8-oxoG site, so that in oligomer **4** the A:T quadruplet (A, adenine) behaves as an almost insurmountable barrier for HT,^4^ whereas the same structural motif acts as an efficient shuttle in the ds-G(T)_4_GGG oligomer, for which an experimental *P*_GGG_/*P*_G_ ratio of 3.5 ± 0.5 was observed.^3^

Modeling charge transport in **4** as an incoherent hopping between pairwise resonant nucleobases leads to a rate constant for crossing the T_4_ bridge of ca. 50 ps^–1^, obviously the same as for ds-G(T)_4_GGG. In contrast, by assuming that solvent activation leads the donor site to be in resonance with all the single G’s on both sides of the T_4_ bridge, a reasonable assumption inasmuch as *in situ* hole site energies of all G’s in the gas phase would fall within a range of a few millielectronvolts, well within vibronic broadening, the hole-transfer dynamics changes significantly, as already observed for oligomer **2**. Predicted time-dependent hole populations for the case in which the hole is initially localized on the G bonded to the T bridge are reported in Figure 4. Within the first 50 ps, the hole mainly bounces among the nearest G sites, without crossing the A:T quadruplet. Appreciable hole population beyond the T bridge is predicted at significantly longer times than in ds-G(T)_4_GGG: at *t* = 150 ps, hole population beyond the T bridge grows up to 50%, beginning to oscillate between the G’s located beyond the T bridge, while hole localization on the G adjacent to the 8-oxoG occurs at still longer time, ca. 250 ps (see the Supporting Information). The results of quantum dynamics simulations for different initial conditions are reported in the Supporting Information: independent of initial conditions, transition times for the hole crossing the T quadruplet in **4** are much longer than in ds-G(T)_4_GGG.

**Figure 4:**
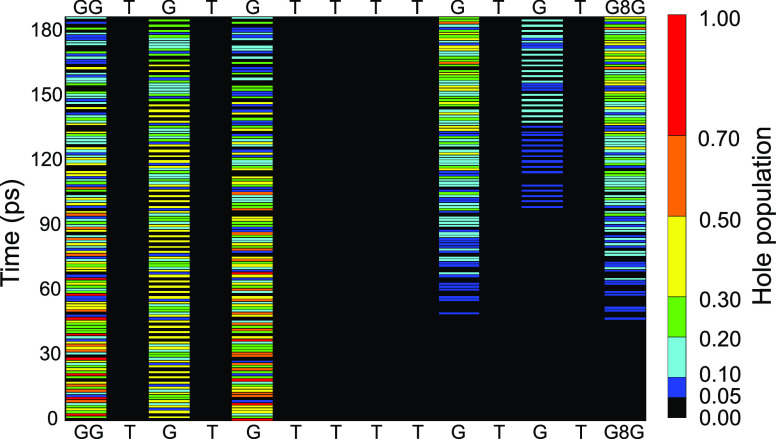
Time evolution of hole population in GGTG_1_TG_2_TTTTG_3_TG_4_TG_5_8G. At *t* = 0 the hole is fully localized on G_2_, which is in electronic resonance with G_3_. Populations are indicated by different color bars (color palette shown on the right side). Computations include both intrastrand and interstrand pathways, including vibronic states of the T quadruplet and of the A quadruplet (whose negligible populations are not shown for clarity) of the complementary strand.

The peculiarity of oligomer **4** is in the fact that several nucleobases can be brought in resonance with the donor site, making it possible that the involvement in dynamics of different indistinguishable paths all contribute to hole localization on the trap site. All those indistiguishable paths—the direct one from any donor which the hole is initially localized on and the several undirect ones, going through any other resonant G’s—concur with the tunneling toward the trap sites, giving rise to interference among probability amplitudes, which significantly affects the overall rate of crossing the T bridge. It is not possible to analyze in further detail the dynamics of the system; any attempt to distinguish among paths would bring back the alternative model of single pairwise resonances. Thus, one can only affirm that when several nucleobases are involved in dynamics the final outcome is different than that observed for a single resonant pair. Hole dynamics depends on eigenvectors, and therefore, a possible alternative way of looking at the phenomenon could be that of tracing back interference among probability amplitudes to the changes in the degree of delocalizations of vibronic eigenvectors in going from the single pairwise resonance to the case of all resonant G’s. In other words, the vibronic eigenstates, once delocalized on several donor species, could exhibit a lower overlap with the states of the T_*n*_ barrier, in comparison to the more localized eigenstate of a single G. (We thank one of the reviewers for suggesting this possibility to us.) Of course that would require the diagonalization of the whole Hamiltonian matrix, which in the present case is unaffordable for the large size of the Hilbert space used in dynamics.

The distributions of oxidative damages predicted by the multistep HT mechanism for the incoherent multihopping mechanism, in which each possible hole path is considered with its own rate (probability), independent of the existence of alternative indistinguishable paths, lead to a high percentage (25%) of oxidative damage at 8-oxoG (see Figure 3d, **4**, left panel). In contrast, the coherent hole transport mechanism, for which HT rates are extracted from quantum dynamics simulations which include all possible paths at once, yields a percentage of oxidative damage at the 8-oxoG of ∼0.1% (see Figure 3d, **4**, right panel), in agreement with experimental results.

The appearance of coherent effects on macroscopic yield ratios could appear somewhat surprising, especially because irreversible damage at nucleobases occurs on a much longer time scale than coherent hole motion. Coherent oscillations are indeed rapidly destroyed by fast solvent deactivation and that, in some cases, can lead to the formation of an equilibrium hole population. That occurs when coherent effects are modest, even though observable, as in the cases of oligomers **2** and **3**. For oligomer **4**, the situation is very different, and coherent effects are crucial; they cause the formation of two different spatial regions, a kinetically allowed region, within which a quasi-equilibrium population is reached on comparatively longer time scales and an almost “kinetically forbidden” region, in which hole populations are always too low for establishing an equilibrium regime. Thus, in oligomer **4** the system is under complete kinetic control. The separation into the two regions occurs on hole-transfer time scales, and therefore, hole trapping probabilities, which in turn determine the final oxidative damage yields on each nucleobase, actually depend on coherent oscillations. The key role played by coherent effects in charge transport along molecular wires has been already recognized in previous works: the flickering resonance^20^ and the unfurling^21^ mechanisms are both based on the assumption that transient degeneracy among different redox species triggers coherent charge motion. It has been shown that coherent effects can account both for the exponential decay of charge-transfer rates in the short-range regime^20,21,37^ and for almost distance-independent rates in the long-range regime.^21^ The mechanistic picture proposed here shares many common points with those mechanisms but adds another important feature: coherent effects can manifest themselves even in the presence of fast dephasing mechanisms.

In summary, we have presented a mechanistic picture of hole transfer in DNA which, taking the role of the environment into account phenomenologically, is able to reconcile apparently discordant experimental data, leading to predicted distributions of oxidative damages at DNA nucleobases in excellent agreement with experimental results. It emerges that in such complex systems, in which charge transport occurs through a manifold of elementary hole-transfer processes regulated by random environmental motions, quantum coherence, i.e., interference among probability amplitudes of indistinguishable paths, plays such a significant role that its underlying presence can be detected at the macroscopic level in the distribution of the final products of charge transport, possibly opening new experimental routes for a better understanding of the effects of quantum coherence in chemistry.^38−41^

## Methods

Many approaches have been developed to solve TDSE for systems where a high number of electronic states are coupled to several nuclear degrees of freedom;^42,43^ here, we employ a simple yet efficient approach in which numerical solution of the TDSE is carried out by using an orthogonalized Krylov subspace method and the most relevant nuclear coordinates coupled to electron hole motion are chosen as those showing the highest equilibrium position displacements upon changing electronic state.^27,44^ Indeed, all the vibrational modes whose equilibrium positions are not affected by hole motion can be kept frozen in their initial quantum state, because changes in quantum numbers would make the Franck–Condon integrals, which determine the couplings with the initial state, vanishingly small (see the Supporting Information). To further lower the computational burden, the entire Hilbert space is partitioned into sets of subspaces, differing in the number of vibrations which are allowed to be simultaneously excited. This partition stems from the observation that the larger the number of simultaneously excited modes, the smaller the relative Franck–Condon integrals, which in our methodology are directly proportional to the coupling between two vibronic states. Thus, it is expected that the effect of states with a significant number of simultaneously excited vibrations on the overall dynamics will be only marginal. Numerical convergence is checked by iteratively increasing the number of subspaces included in the computations until no significant variations of the properties of interest (i.e., electronic population and transition times) are observed (convergence tests are reported in the Supporting Information). This heuristic approach allows a significant restriction of the active space of the problem and the associated numerical complexity, while still retaining the most important features of the dynamical behavior of the system. Only the strand on which the charge is initially injected has been considered in dynamics, except for ds-G(T)_*n*_GGG with *n* > 3 of the first set and for oligomer **4** of the second set of oligomers (Figure 1), because the latter oligomers contain in the complementary strand A multiplets, which possess a hole site energy low enough to play a role in hole transfer.^29^ In those specific cases, hole-transfer dynamics have been performed by considering the whole double strands.

## Supporting Information Available

Detailed description of the model Hamiltonian; strategy for the selection of the vibrational states included in quantum dynamics simulations; hole site energies and electron couplings (Table S1); wavenumbers, intramolecular reorganization energies, and equilibrium position displacements (Table S2); convergence tests for different Hilbert subspaces (Figure S1); quantum dynamics populations for ds-G(T)_*n*_GGG oligomers (Figure S2) and oligomers 2 and 4 (Figures S3 and S4–S7); kinetic schemes together with rate constants for simulating charge transfer in all the oligomers of Figure 1 (PDF)
