# Supplementary material for: Coherent Effects in Charge Transport in Molecular Wires: Toward a Unifying Picture of Long-Range Hole Transfer in DNA
Source: J Phys Chem Lett. 2020 Aug 24;11(18):7769–75. doi: 10.1021/acs.jpclett.0c01996 (PMC8154848; doi:10.1021/acs.jpclett.0c01996)
Supplement: Supplementary file 1 — jz0c01996_si_001.pdf [file jz0c01996_si_001.pdf]

Supporting Information: Coherent Effects in Charge Transport in Molecular Wires: Toward a Unifying Picture of Long Range Hole Transfer in DNA

Alessandro Landi, Amedeo Capobianco, and Andrea Peluso*

Dipartimento di Chimica e Biologia

Università di Salerno, I-84084 Fisciano, Salerno, Italy

E-mail: apeluso@unisa.it

The Model Hamiltonian

Let us consider a supramolecular assembly constituted by L weakly interacting units on which a charge, an electron or a hole, has been injected. The electronic wavefunction can be expanded over a basis of L diabatic states, each of them corresponding to the charge fully localized on one molecular unit. Since the intermolecular electronic couplings are much smaller than electronic energies, each electronic diabatic state $|\ell\rangle$ can be well represented by the direct product of the eigenstates of the non-interacting molecular units:

$$|\ell\rangle = |\ell_C\rangle \prod_{i \neq \ell}^{L} |i_N\rangle, \tag{1}$$

where, $|\ell\rangle$ is the electronic state in which the additional charge is entirely localized on the molecular unit ℓ, while subscripts C and N indicate the charged and neutral states, respectively. The electronic states $|i_X\rangle$ are the eigenfunctions of the electronic Hamiltonian operators of the individual i-th molecular unit in its redox state $X = C, N$:

$$\mathcal{H}_{iX}^{(\mathrm{el})}|i_X\rangle = U_{iX}(\mathbf{Q}_{iX})|i_X\rangle, \quad X = C,\ N; \quad i = 1,\ 2, \ldots, L. \tag{2}$$

where $U_{iX}(\mathbf{Q}_{iX})$ is the electronic energy and $\mathbf{Q}_{iX}$ are the normal coordinates of the i-th molecular units.

In the following we adopt the harmonic approximation for the U_{iX}'s:

$$U_{iX} = E_{iX}^{0} + \frac{1}{2}\mathbf{Q}_{iX}^{\dagger}\boldsymbol{\omega}_{iX}^{2}\mathbf{Q}_{iX}, \tag{3}$$

where E_{iX}^{0} is the electronic energy of $|i_X\rangle$ at its equilibrium geometry, while ω_{iX} is the diagonal matrix of the vibrational frequencies.

We write the wavefunction as a Born-Oppenheimer product $|\ell, \bar{v}_\ell\rangle$, where $\bar{v}_\ell$ is the multi-index vector of the vibrational quantum number associated to $|\ell\rangle$. Within this framework, the Hamiltonian operator of the whole system can be written as:[S1,S2]

$$\mathcal{H} = \sum_{\ell,\bar{v}_\ell} E_{\bar{v}_\ell}|\ell, \bar{v}_\ell\rangle\langle\ell, \bar{v}_\ell| + \sum_{\ell,m,\bar{v}_\ell,\bar{v}_m,i}\left(H_{\ell m} + \frac{\partial H_{\ell m}}{\partial Q_i}Q_i\right)|\ell, \bar{v}_\ell\rangle\langle m, \bar{v}_m| + \mathrm{c.c.} \tag{4}$$

where $E_{\bar{v}_\ell}$ is the vibronic energy of the state $|\ell, \bar{v}_\ell\rangle$ and $H_{\ell m}$ is the electronic coupling term. $\frac{\partial H_{\ell m}}{\partial Q_i} = 0.1$ eV/Åhas been introduced to take into account the weak dependence of the electronic coupling terms on the nuclear coordinates, especially the one due to the inter-bases vibrations.[S3]

The time-dependent wavefunction is then written as:

$$|\Psi(t)\rangle = \sum_{\ell,\bar{v}_\ell} C_{\ell,\bar{v}_\ell}(t)|\ell, \bar{v}_\ell\rangle \tag{5}$$

where the time-dependent expansion coefficients can be computed by solving the time-dependent Schrödinger equation

$$
\mathrm{i}\hbar \begin{pmatrix} \dot{\mathbf{C}}^{(1)}_{\bar{v}_1} \\ \cdot \\ \cdot \\ \dot{\mathbf{C}}^{(L)}_{\bar{v}_L} \end{pmatrix} = \begin{pmatrix} \mathbf{H}_{11} & \mathbf{H}_{12} & . & . & \mathbf{H}_{1L} \\ & . & . & . & \\ & . & . & . & \\ \mathbf{H}^{\dagger}_{1L} & \mathbf{H}^{\dagger}_{2L} & . & . & \mathbf{H}_{LL} \end{pmatrix} \begin{pmatrix} \mathbf{C}^{(1)}_{\bar{v}_1} \\ \cdot \\ \cdot \\ \mathbf{C}^{(L)}_{\bar{v}_L} \end{pmatrix}, \tag{6}
$$

with initial conditions specifying the initial state of the system. In Eq. 6, each $\mathbf{H}_{\ell m}$ and $\dot{\mathbf{C}}^{(\ell)}_{\bar{v}_\ell}$ is a matrix itself, whose size depends on the vibrational basis sets chosen for the electronic states ℓ and m.

Several strategies to reduce the size of the vibronic basis set have been proposed in the literature.[S4] The approach we use here is based on the idea (applied in the field of molecular spectroscopy by Jankowiack[S5] and Santoro,[S6] but used for the first time in quantum dynamics by our group[S7]) of partitioning the entire Hilbert space H_s spanned by the Hamiltonian of Eq. 5 as:

$$
H = \bigcup_s h_s
$$

where h_s is the Hilbert space spanned by the states in which only s vibrations are simultaneously excited.

Following this approach, the wavefunction of Eq. 5 is then written as sum of all the contributions belonging to each subspace and the expansion is truncated to a certain value of s:

$$
\big|\Psi(t)\big\rangle = \sum_\ell \left[C_{\ell,0}(t)\big|\ell,0\big\rangle + \sum_i^N \sum_{v_i} C_{\ell,v_i}(t)\big|\ell,v_i\big\rangle + \sum_{ij}^{\binom{N}{2}} \sum_{v_i,v_j} C_{\ell,v_i,v_j}(t)\big|\ell,v_i,v_j\big\rangle + \dots \right] \tag{7}
$$

where $\big|\ell,0\big\rangle$ is the vibrational ground state of the ℓ-th electronic state, while $\big|\ell,v_i\big\rangle$ and

$|\ell, v_i, v_j\rangle$ denote states belonging to Hilbert subspaces in which only one and two vibrational degrees of freedom are simultaneously excited. The indexes i and j run on the whole manifold of the N active normal modes of the ℓ-th electronic state and v_i runs on vibrational quantum numbers of the i-th mode.

This partition of the Hilbert space is based on the observation that Franck-Condon (FC) integrals usually become increasingly smaller as the number of simultaneously excited modes increases.[S5,S6,S8] Since the couplings between two vibronic states are proportional to the corresponding FC integrals, vibronic states which are expected to be more effectively coupled to electronic motion are those included in the subspaces with lower dimensions, i.e. $s = 1 - 3$, whereas subspaces with a higher number of simultaneously excited vibrations should hopefully play only a minor role on the overall dynamics. In fact, it has been shown[S9] that, in the calculation of spectral band shapes, including only states belonging to $s = 1 - 3$ subspaces allows for recovering about 85% of the intensity of the whole spectrum at 0 K.

The nearest neighbor approximation has been used throughout this work.

Hole site energies and electronic coupling parameters

Adopted hole site energies and intrastrand electronic couplings for stacked nucleobases, are reported in Table S1. The inter-strand G-A electronic coupling element to 0.012 eV, as in previous works.[S11,S12]

Table S1: Hole site energies (E_Y, eV, relative to the G/G$^+$ pair) and electronic coupling parameters for stacked base pairs (H_{YX}, eV). All values are extracted from ref. S10.

Y	E_Y	H_{YG}	H_{YA}	H_{YC}	H_{YT}
G	0.00	0.09	0.16	0.23	0.16
A	0.43	0.12	0.27	0.15	0.09
C	0.68	0.25	0.18	0.12	0.13
T	0.70	0.13	0.08	0.09	0.12

Selection of active normal modes

The most important issue when dealing with quantum dynamics simulations is the selection of the vibrational states to be used in the time evolution. Two main factors determine the relevance of a vibronic state in the dynamics of the system: its energy and its couplings with all the states that can be populated in the time evolution of the system. Since the coupling can also act indirectly (that is, mediated by a third state, possibly at higher energy), a mere selection of vibronic states falling into a small energy range would be ineffective. In the computational scheme used here, the problem of indirect couplings is somewhat relieved by using a basis of harmonic vibrational states for each electronic state, so that all the $\mathbf{H}_{ii}$ of Eq. 6 are diagonal matrices. With that basis set, the most coupled modes can be selected by projecting the normal modes of one electronic state into those of the other, by the affine Duschinsky's transformation;[S13]

$$\mathbf{Q}_\ell = \mathbf{J}\mathbf{Q}_m + \mathbf{K}, \tag{8}$$

where $\mathbf{Q}_\ell$ and $\mathbf{Q}_m$ vectors collect the normal mode coordinates of $|\ell\rangle$ and $|m\rangle$; $\mathbf{J}$ is the rotation matrix, and $\mathbf{K}$ the displacement vector. Those quantities can be determined once the equilibrium geometries and the normal modes of the two electronic states are known. Duschinsky's affine transformation is also the starting point for evaluating multidimensional Franck-Condon integrals.[S14,S15]

The "active" modes in dynamics thus correspond to the normal coordinates undergoing the highest mixing and equilibrium position displacements in passing from $|\ell\rangle$ to $|m\rangle$ electronic states. Only those modes that are either displaced or mixed with other modes by the electronic transition $|\ell\rangle \rightarrow |m\rangle$ can change their quantum numbers during the evolution of the system. All other modes can be kept frozen in their initial quantum state, because changes in quantum numbers would make the Franck-Condon integrals, i.e. the couplings with the initial state, vanishingly small.

Noteworthy, only the normal modes of the DNA units actually exchanging an electron

will really matter; the modes of all the other units turn out to be unchanged, inasmuch as the vibrational wavefunction is factorized (cf. Eq. 1).

Table S2: Wavenumbers (ω, cm^{-1}), intramolecular reorganization energies (E_r, cm^{-1}), and equilibrium position displacements ($\mathbf{K}$, absolute values Å $uma^{1/2}$) of the most displaced normal coordinates of G/G^+, A/A^+, C/C^+ and T/T^+ redox pairs. Electronic calculation at the PCM(water)B3LYP/6-311++G(d,p) level.

G/G^+			A/A^+			C/C^+			T/T^+		
ω	$E_r{}^a$	$\mathbf{K}$	ω	$E_r{}^a$	$\mathbf{K}$	ω	$E_r{}^a$	$\mathbf{K}$	ω	$E_r{}^a$	$\mathbf{K}$
338	76	0.21	724	88	0.106	348	27	0.39	394	90	0.20
435	223	0.28	1328	83	0.056	500	32	0.36	536	127	0.17
477	80	0.15	1143	83	0.065	574	187	0.81	709	93	0.11
521	166	0.20	1353	204	0.087	1043	123	0.49	1320	449	0.13
528	78	0.14	1367	84	0.055	1244	194	0.56	1363	139	0.071
1230	75	0.058	1510	424	0.11	1277	87	0.37	1590	675	0.13
1366	85	0.055	1622	109	0.053	1361	107	0.40			
1403	121	0.064	1639	167	0.065	1506	87	0.34			
1435	90	0.054									
1477	259	0.089									
1526	80	0.048									
1639	488	0.11									
1742	160	0.06									
$E_r{}^b$	2249			1658			1121			1937	

^{a} Computed by harmonic approximation from $\mathbf{K}$ components. ^{b}From electronic computations.

The components of the $\mathbf{K}$ vector for the most displaced and mixed modes of G/G^+, A/A^+, C/C^+ and T/T^+ redox pairs are reported in Table S2, together with the total reorganization energy of the individual nucleobases from DFT calculations in water solution. More than 80% of the total reorganization energy is recovered by using 13 modes for G/G^+ half pair, 6 for T/T^+, and 8 for A/A^+ and C/C^+, so that inclusion of only these modes should provide a qualitatively correct picture of hole transport in the short oligonucleotides considered in this work.

Convergence tests

The partition of eq. 7, together with the selection of the active modes described in previous section, allows for a substantial reduction of the size of the basis set thus making the computations feasible. If each mode is allowed to have Z basis functions, then c simultaneously

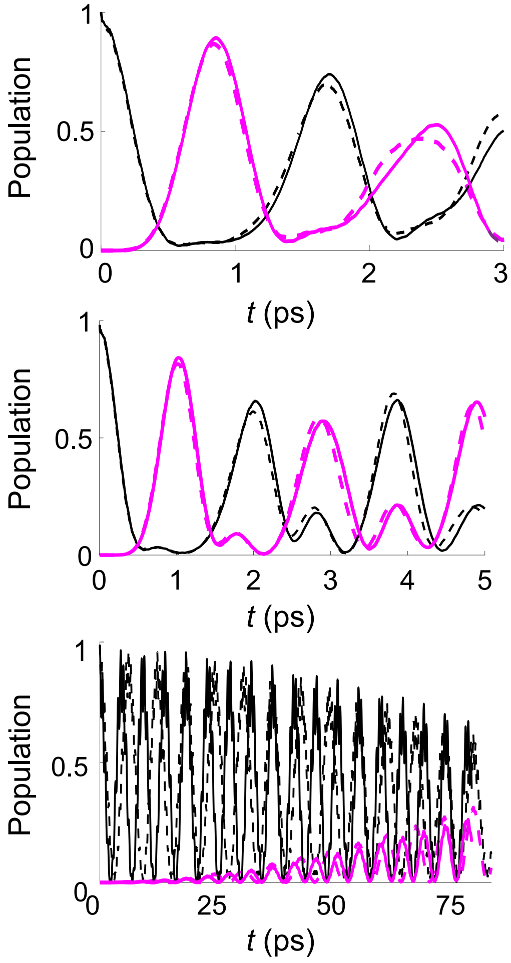

Figure S1: Comparison of time-dependent populations using a Hilbert subspace with up to 2 simultaneously excited vibrations (full lines) or up to 3 simultaneously excited vibrations (dashed lines), assuming electronic degeneracy for all the G sites for oligomers **1** (top), **2** (center) and **4** (bottom). Only the populations of the $3'$ (magenta) and $5'$ (black) Gs are shown for clarity.

excited modes will introduce Z^c states to be considered in dynamics, amounting to $\binom{N}{c}$ combinations, so that S_c space comprises $\binom{N}{c}Z^c$ states. Thus, $c = 1$ gives rises to N possible combinations and NZ basis functions, $c = 2$ originates $\binom{N}{2}Z^2$ states and so on. In a system with $N = 50$ and $Z = 10$ the full tensor product basis set (i.e. considering $c = N$) will have a

size amounting to 10^{50}, which is far beyond the limit of any modern numerical methodology. By assuming that only the combinations up to $c = 3$ are relevant, the size of the basis set for the same system will be $\approx 10^7$, which is numerically treatable. Furthermore, we have devised a grouping procedure in which the number of simultaneously excited modes is increased only for a restricted subset of vibrations. Numerical convergence can then be tested by letting c vary until no significant variations of the properties of interest (i.e. electronic population or coherence) are observed.

Indeed, an inspection of figure S1, where results of quantum dynamics simulations carried out with $c = 2$ and $c = 3$ are compared, shows that a satisfactory convergence is reached by including in calculations subspaces up to order $c = 3$. Differences are mainly related to the populations, while transition times, the information we need to build up the kinetic scheme, do not exhibit significant changes.

Quantum Dynamics simulations

In this section we report the results of quantum dynamics simulations performed on the oligomers discussed in the main text.

Set 1 In Figure S2 the yield ratios of oxidative damage in ds-G(T)$_n$GGG oligomers are reported together with their experimental counterparts. The results are very similar to the previous ones and have already been extensively discussed in ref. S12.

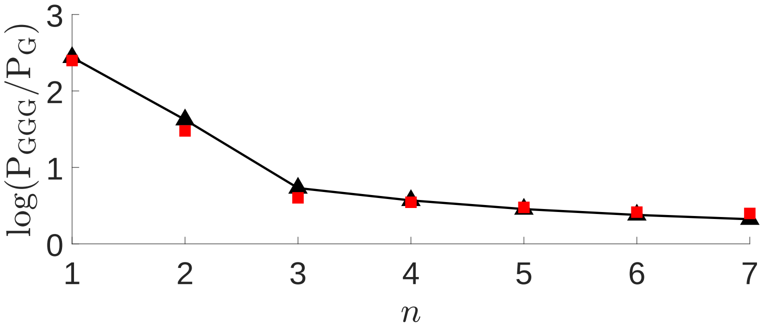

Figure S2: Predicted (black triangles) and experimental[S16] (red squares) yield ratios for the HT in ds-G(T)$_n$GGG as a function of the number of bases separating the donor and the acceptor G sites.

Oligomer 1

In figure S3 we report the quantum dynamics population as a function of time for the oligomer **1** for different resonance conditions; as discussed in the main text, hole transfer among two Gs separated by a single T bridge is very fast, of the order of a few tenths of picoseconds, and grows to 5 and 15 ps when resonant Gs are separated by an off-resonant TGT and TGTGT bridge, respectively

Oligomer 2

In figure S4 we report the quantum dynamics population as a function of time for the oligomer **2** assuming that the donor and acceptor GG steps are in electronic resonance, or that the initial 3′-GG step is in in electronic resonance with the fourth single G, resonant sites being separated by a off-resonance TGTGTGT bridge. As discussed in the main text direct hole transfer between G doublet and the farthest single G occurs in ca. 50 ps (panel

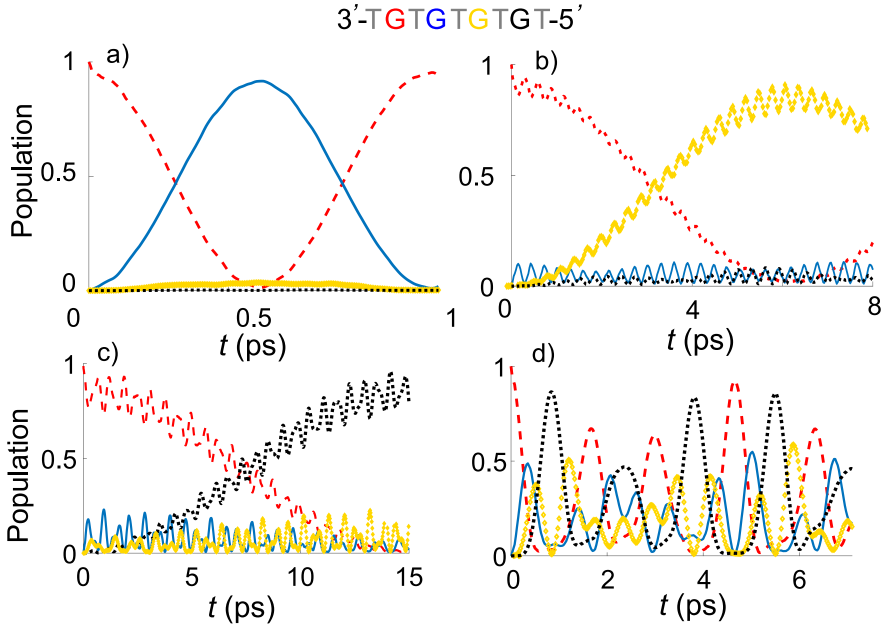

Figure S3: Quantum dynamics population as a function of time for the oligomer **1** $TG_1TG_2TG_3TG_4T$: (a) two nearest G (i.e. G_1 and G_2) in electronic resonance; (b) resonant Gs separated by a off resonance TGT bridge (i.e. G_1 and G_3 in resonance); (c) resonant Gs separated by a off resonance TGTGT bridge (i.e. G_1 and G_4 in resonance); all Gs in electronic resonance (d). The last two cases are also reported in the main text

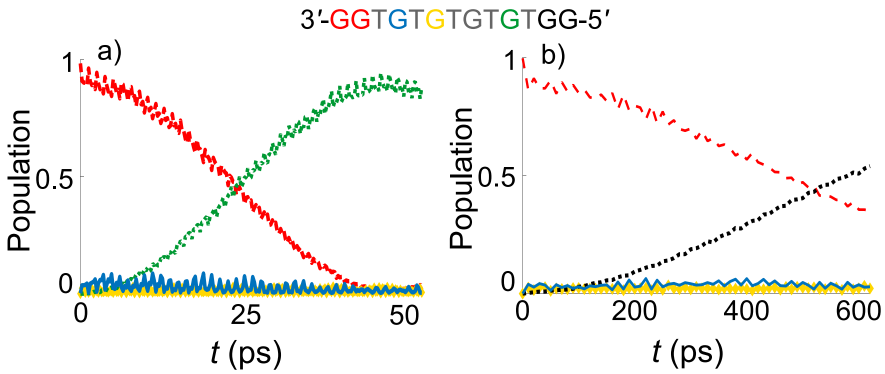

Figure S4: Quantum dynamics population as a function of time for the oligomer GGTGTGTGT-GTGG. a) GG step in electronic resonance with the fourth single G, separated by a off-resonance TGTGTGT bridge; b) resonant GG steps separated by a off resonance TGTGTGTGT bridge. Gs showing negligible populations in all the time interval considered are not shown for clarity.

a), while direct hole transfer between G doublets requires more than 0.5 ns (panel b).

Oligomer 4

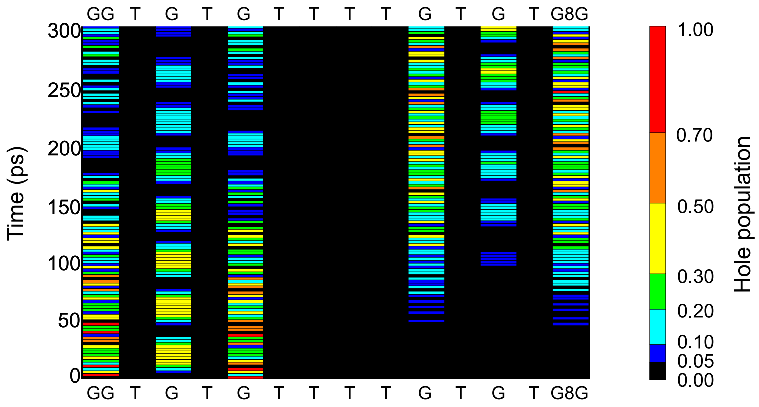

Figure S5: Time evolution in $GGTG_1TG_2TTTTG_3TG_4TG_58G$ for the hole initially localized on G_2. All the guanine sites are assumed to be in electronic degeneracy. As outlined by the legend in the figure, a red square indicates that at this time the damage fraction on the molecule is between 0.7 and 1.0, etc. Slight differences in the coloring scheme with respect to figure 4 in the main text are due to the different time sampling used.

In figure S5, the quantum dynamics simulations for oligomer **4** starting with the hole fully localized on the G just preceding the T bridge is reported (for longer times than shown in the main text). Complete hole localization on the G adjacent to the 8-oxoG occurs after about 250 ps.

Other dynamics simulations have been considered with different initial conditions and/or electronic degeneracy among any G/GG of the first tract before the T quadruplet and any G's of the second tract.

In figure S6 we report the quantum dynamics simulations for oligomer **4** with all the Gs in electronic resonance, as in figure S5, but starting from the GG step on the left. As expected, since the HT between the Gs ahead of the T4 bridge is much faster, the population evolution for the Gs beyond the T_4 step is quite similar to the case in figure S5. Finally, when considering different electronic degeneracy among any G/GG step in oligomer **4**, the population dynamics change slightly, but hole transfer towards the final 8-oxoG step is predicted to occur on similar timescales (see figure S7).

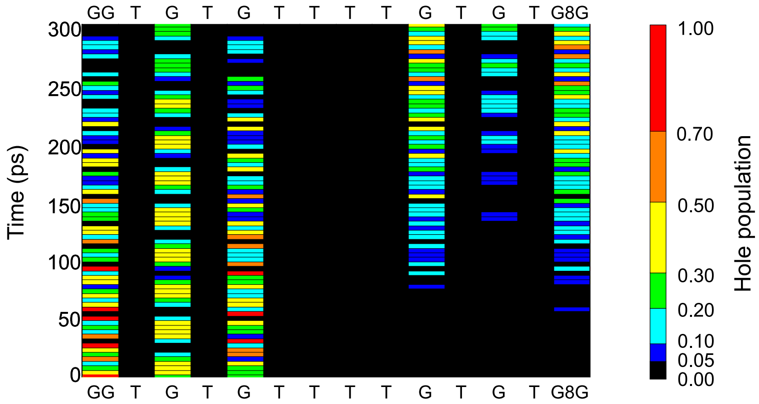

Figure S6: Time evolution in GGTGTGTTTTGTGT8G for the hole initially localized on the left GG. All the guanine sites are assumed to be in electronic degeneracy.

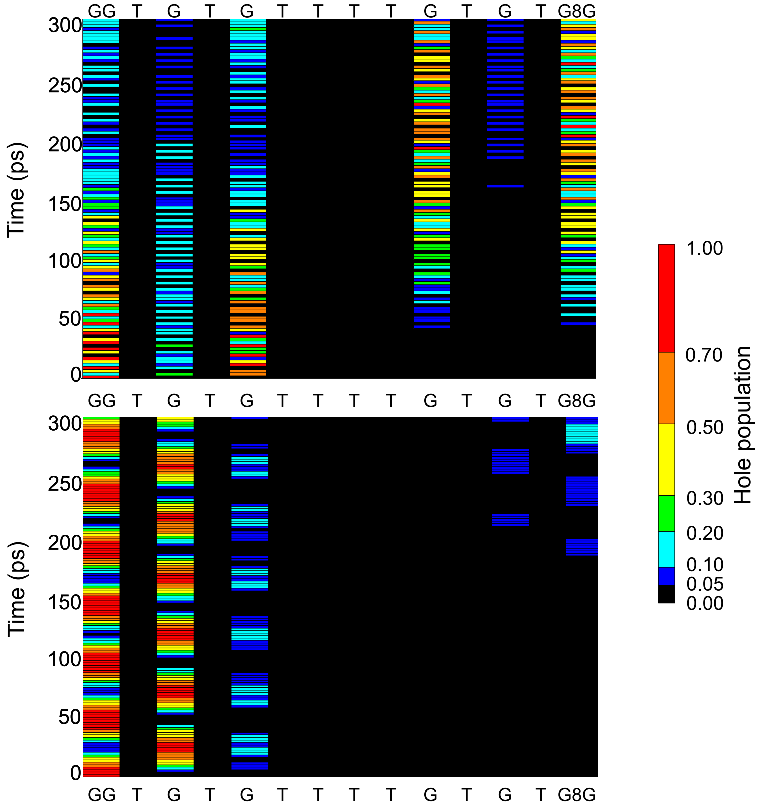

Figure S7: Time evolution of hole population in $GGTG_1TG_2TTTTG_3TG_4TG_58G$; the hole is initially localized on the left GG. Different degeneracy among the guanine sites are considered: (top) GG, G_2, G_3 and G_58G in degeneracy; (bottom) GG, G_1, G_4 and G_58G

Generalized kinetic schemes

Here we report all the possible charge transfer paths for the different oligomers belonging to set 2, together with the kinetic constants (in s^{-1}) discussed in the main text. Following the

conventions used in the main text, A^* denotes the activated species; when more than one activated species is possible, e.g. for GG steps, which can be in electronic resonance with a single G or with another GG step, the different possibilities are differentiated by adding a † symbol (e.g. $GG_1^{*,\dagger}$).

Oligomer 1

$$\mathrm{TG_1TG_2TG_3TG_4T}$$

$$
\begin{aligned}
&\mathrm{G}_i \underset{10^{13}}{\overset{10^{10}}{\rightleftharpoons}} \mathrm{G}_i^* \qquad i=1,4 \\
&\mathrm{G_i} \overset{1\times10^{7}}{\rightarrow} \mathrm{P_{G_i}} \qquad i=1,4 \\
&\mathrm{G}_1^* \underset{2.7\times10^{12}}{\overset{2.7\times10^{12}}{\rightleftharpoons}} \mathrm{G}_2^* \\
&\mathrm{G}_1^* \underset{2.0\times10^{11}}{\overset{2.0\times10^{11}}{\rightleftharpoons}} \mathrm{G}_3^* \\
&\mathrm{G}_1^* \underset{6.7\times10^{10}}{\overset{6.7\times10^{10}}{\rightleftharpoons}} \mathrm{G}_4^* \\
&\mathrm{G}_2^* \underset{2.7\times10^{12}}{\overset{2.7\times10^{12}}{\rightleftharpoons}} \mathrm{G}_3^* \\
&\mathrm{G}_2^* \underset{2.0\times10^{11}}{\overset{2.0\times10^{11}}{\rightleftharpoons}} \mathrm{G}_4^* \\
&\mathrm{G}_3^* \underset{2.7\times10^{12}}{\overset{2.7\times10^{12}}{\rightleftharpoons}} \mathrm{G}_4^*
\end{aligned}
\tag{9}
$$

Oligomer 2

$$\mathrm{GG_1TG_1TG_2TG_3TG_4TGG_2}$$

$$
\begin{aligned}
&\mathrm{G}_i \underset{10^{13}}{\overset{10^{10}}{\rightleftharpoons}} \mathrm{G}_i^* \qquad i=1,4\\
&\mathrm{G}_i \overset{1\times10^{7}}{\rightarrow} \mathrm{P}_{\mathrm{G}_i} \qquad i=1,4\\
&\mathrm{GG}_i \underset{10^{13}}{\overset{4.9\times10^{8}}{\rightleftharpoons}} \mathrm{GG}_\mathrm{i}^* \qquad i=1,2 &&\mathrm{G}_1^* \underset{2.7\times10^{12}}{\overset{2.7\times10^{12}}{\rightleftharpoons}} \mathrm{G}_2^*\\
&\mathrm{GG}_i \underset{10^{13}}{\overset{10^{10}}{\rightleftharpoons}} \mathrm{GG}_i^{*,\dagger} \qquad i=1,2 &&\mathrm{G}_1^* \underset{2.0\times10^{11}}{\overset{2.0\times10^{11}}{\rightleftharpoons}} \mathrm{G}_3^*\\
&\mathrm{GG}_i \overset{3\times10^{6}}{\rightarrow} \mathrm{P}_{\mathrm{GG}_i} \qquad i=1,2 &&\mathrm{G}_1^* \underset{6.7\times10^{10}}{\overset{6.7\times10^{10}}{\rightleftharpoons}} \mathrm{G}_4^*\\
&\mathrm{GG}_1^* \underset{2.7\times10^{12}}{\overset{2.7\times10^{12}}{\rightleftharpoons}} \mathrm{G}_1^* &&\mathrm{G}_1^* \underset{2.0\times10^{10}}{\overset{2.0\times10^{10}}{\rightleftharpoons}} \mathrm{GG}_2^*\\
&\mathrm{GG}_1^* \underset{2.0\times10^{11}}{\overset{2.0\times10^{11}}{\rightleftharpoons}} \mathrm{G}_2^* &&\mathrm{G}_2^* \underset{2.7\times10^{12}}{\overset{2.7\times10^{12}}{\rightleftharpoons}} \mathrm{G}_3^*\\
&\mathrm{GG}_1^* \underset{6.7\times10^{10}}{\overset{6.7\times10^{10}}{\rightleftharpoons}} \mathrm{G}_3^* &&\mathrm{G}_2^* \underset{2.0\times10^{11}}{\overset{2.0\times10^{11}}{\rightleftharpoons}} \mathrm{G}_4^*\\
&\mathrm{GG}_1^* \underset{2.0\times10^{10}}{\overset{2.0\times10^{10}}{\rightleftharpoons}} \mathrm{G}_4^* &&\mathrm{G}_2^* \underset{6.7\times10^{10}}{\overset{6.7\times10^{10}}{\rightleftharpoons}} \mathrm{GG}_2^*\\
&\mathrm{GG}_1^{*,\dagger} \underset{1.5\times10^{9}}{\overset{1.5\times10^{9}}{\rightleftharpoons}} \mathrm{GG}_2^{*,\dagger} &&\mathrm{G}_3^* \underset{2.7\times10^{12}}{\overset{2.7\times10^{12}}{\rightleftharpoons}} \mathrm{G}_4^*\\
& &&\mathrm{G}_3^* \underset{2.0\times10^{11}}{\overset{2.0\times10^{11}}{\rightleftharpoons}} \mathrm{GG}_2^*\\
& &&\mathrm{G}_4^* \underset{2.7\times10^{12}}{\overset{2.7\times10^{12}}{\rightleftharpoons}} \mathrm{GG}_2^*
\end{aligned}
\tag{10}
$$

Oligomer 3

$$\mathrm{GGTG_1TG_2TG_3TG_4TG_58G}$$

$$\mathrm{GG} \underset{10^{13}}{\overset{4.9\times10^{8}}{\rightleftharpoons}} \mathrm{GG}^{*}$$

$$\mathrm{GG} \underset{10^{13}}{\overset{10^{10}}{\rightleftharpoons}} \mathrm{GG}^{*,\dagger}$$

$$\mathrm{GG} \overset{3\times10^{6}}{\rightarrow} \mathrm{P_{GG}}$$

$$\mathrm{GG}^{*} \underset{2.7\times10^{12}}{\overset{2.7\times10^{12}}{\rightleftharpoons}} \mathrm{G}_1^{*}$$

$$\mathrm{GG}^{*} \underset{2.0\times10^{11}}{\overset{2.0\times10^{11}}{\rightleftharpoons}} \mathrm{G}_2^{*}$$

$$\mathrm{GG}^{*} \underset{6.7\times10^{10}}{\overset{6.7\times10^{10}}{\rightleftharpoons}} \mathrm{G}_3^{*}$$

$$\mathrm{GG}^{*} \underset{2.0\times10^{10}}{\overset{2.0\times10^{10}}{\rightleftharpoons}} \mathrm{G}_4^{*}$$

$$\mathrm{GG}^{*} \underset{1.0\times10^{10}}{\overset{1.0\times10^{10}}{\rightleftharpoons}} \mathrm{G}_5^{*}$$

$$\mathrm{G}_i \underset{10^{13}}{\overset{10^{10}}{\rightleftharpoons}} \mathrm{G}_i^{*} \qquad i = 1, 5$$

$$\mathrm{G}_i \overset{1\times10^{7}}{\rightarrow} \mathrm{P}_{\mathrm{G}_i} \qquad i = 1, 5$$

$$\mathrm{G}_1^{*} \underset{2.7\times10^{12}}{\overset{2.7\times10^{12}}{\rightleftharpoons}} \mathrm{G}_2^{*}$$

$$\mathrm{G}_1^{*} \underset{2.0\times10^{11}}{\overset{2.0\times10^{11}}{\rightleftharpoons}} \mathrm{G}_3^{*}$$

$$\mathrm{G}_1^{*} \underset{6.7\times10^{10}}{\overset{6.7\times10^{10}}{\rightleftharpoons}} \mathrm{G}_4^{*}$$

$$\mathrm{G}_1^{*} \underset{2.0\times10^{10}}{\overset{2.0\times10^{10}}{\rightleftharpoons}} \mathrm{G}_5^{*}$$

$$\mathrm{G}_2^{*} \underset{2.7\times10^{12}}{\overset{2.7\times10^{12}}{\rightleftharpoons}} \mathrm{G}_3^{*}$$

$$\mathrm{G}_2^{*} \underset{2.0\times10^{11}}{\overset{2.0\times10^{11}}{\rightleftharpoons}} \mathrm{G}_4^{*}$$

$$\mathrm{G}_2^{*} \underset{6.7\times10^{10}}{\overset{6.7\times10^{10}}{\rightleftharpoons}} \mathrm{G}_5^{*}$$

$$\mathrm{G}_3^{*} \underset{2.7\times10^{12}}{\overset{2.7\times10^{12}}{\rightleftharpoons}} \mathrm{G}_4^{*}$$

$$\mathrm{G}_3^{*} \underset{2.0\times10^{11}}{\overset{2.0\times10^{11}}{\rightleftharpoons}} \mathrm{G}_5^{*}$$

$$\mathrm{G}_4^{*} \underset{2.7\times10^{12}}{\overset{2.7\times10^{12}}{\rightleftharpoons}} \mathrm{G}_5^{*}$$

$$\mathrm{G}_5^{*} \overset{10^{13}}{\rightarrow} \mathrm{8G} \tag{11}$$

Oligomer 4

$$\mathrm{GGTG_1TG_2TTTTG_3TG_4TG_58G}$$

$$
\begin{aligned}
&\mathrm{GG} \underset{10^{13}}{\overset{4.9\times10^{8}}{\rightleftharpoons}} \mathrm{GG}^{*} \\
&\mathrm{GG} \underset{10^{13}}{\overset{10^{10}}{\rightleftharpoons}} \mathrm{GG}^{*,\dagger} \\
&\mathrm{GG} \overset{3\times10^{6}}{\rightarrow} \mathrm{P_{GG}} \\
&\mathrm{GG}^{*} \underset{2.7\times10^{12}}{\overset{2.7\times10^{12}}{\rightleftharpoons}} \mathrm{G}_1^{*} \\
&\mathrm{GG}^{*} \underset{2.0\times10^{11}}{\overset{2.0\times10^{11}}{\rightleftharpoons}} \mathrm{G}_2^{*} \\
&\mathrm{GG}^{*} \underset{3.0\times10^{10}}{\overset{3.0\times10^{10}}{\rightleftharpoons}} \mathrm{G}_3^{*} \\
&\mathrm{GG}^{*} \underset{3.0\times10^{10}}{\overset{3.0\times10^{10}}{\rightleftharpoons}} \mathrm{G}_4^{*} \\
&\mathrm{GG}^{*} \underset{3.0\times10^{10}}{\overset{3.0\times10^{10}}{\rightleftharpoons}} \mathrm{G}_5^{*} \\
&\mathrm{G}_i \underset{10^{13}}{\overset{10^{10}}{\rightleftharpoons}} \mathrm{G}_i^{*} \qquad i=1,5 \\
&\mathrm{G}_i \overset{1\times10^{7}}{\rightarrow} \mathrm{P}_{\mathrm{G}_i} \qquad i=1,5 \\
&\mathrm{G}_1^{*} \underset{2.7\times10^{12}}{\overset{2.7\times10^{12}}{\rightleftharpoons}} \mathrm{G}_2^{*} \\
&\mathrm{G}_1^{*} \underset{3.0\times10^{10}}{\overset{3.0\times10^{10}}{\rightleftharpoons}} \mathrm{G}_3^{*} \\
&\mathrm{G}_1^{*} \underset{3.0\times10^{10}}{\overset{3.0\times10^{10}}{\rightleftharpoons}} \mathrm{G}_4^{*} \\
&\mathrm{G}_1^{*} \underset{2.0\times10^{10}}{\overset{2.0\times10^{10}}{\rightleftharpoons}} \mathrm{G}_5^{*} \\
&\mathrm{G}_2^{*} \underset{3.0\times10^{10}}{\overset{3.0\times10^{10}}{\rightleftharpoons}} \mathrm{G}_3^{*} \\
&\mathrm{G}_2^{*} \underset{2.0\times10^{11}}{\overset{2.0\times10^{11}}{\rightleftharpoons}} \mathrm{G}_4^{*} \\
&\mathrm{G}_2^{*} \underset{6.7\times10^{10}}{\overset{6.7\times10^{10}}{\rightleftharpoons}} \mathrm{G}_5^{*} \\
&\mathrm{G}_3^{*} \underset{2.7\times10^{12}}{\overset{2.7\times10^{12}}{\rightleftharpoons}} \mathrm{G}_4^{*} \\
&\mathrm{G}_3^{*} \underset{2.0\times10^{11}}{\overset{2.0\times10^{11}}{\rightleftharpoons}} \mathrm{G}_5^{*} \\
&\mathrm{G}_4^{*} \underset{2.7\times10^{12}}{\overset{2.7\times10^{12}}{\rightleftharpoons}} \mathrm{G}_5^{*} \\
&\mathrm{G}_5^{*} \overset{10^{13}}{\rightarrow} \mathrm{8G}
\end{aligned}
\tag{12}
$$

Computational methods

Equilibrium geometries, normal modes, and vibrational frequencies of nucleobases in their neutral and cationic form were computed at the DFT level by the B3LYP functional with the 6-311++G(d,p) basis set. The unrestricted formalism has been used for doublet electronic states. Solvent (water) effects have been estimated by the polarizable continuum model

(PCM);[S17] the Gaussian09 package has been used for all electronic computations.[S18]

Franck-Condon integrals have been computed by using a development version of the MolFC package,[S19,S20] In all FC calculations, the curvilinear coordinate representation of the normal modes has been adopted to prevent that a large displacement of an angular coordinate could reflect into large shifts of the involved bond distances. That is unavoidable in rectilinear Cartesian coordinates and requires the use of high order anharmonic potentials for its correction.[S21–S24] To further reduce the overall computational costs, the computation of the FC integrals has been carried out by using the separate-mode approximation, which leads to factorization of the multidimensional FC integrals into the product of one-dimensional integrals.[S25] That approximate method for fast FC computations corresponds roughly to neglecting the off diagonal terms of the Duschinsky transformation but taking into account the changes of the vibrational frequencies of the vibrational modes.

The numerical solution of the time-dependent Schrödinger equation has been carried out with an orthogonalized Krylov subspace method.[S4,S26]

References

(S1) M. Bixon, *et al.*, *Proc. Natl. Acad. Sci. USA* **96**, 11713 (1999).

(S2) S. S. Skourtis, S. Mukamel, *Chem. Phys.* **197**, 367 (1995).

(S3) A. Landi, A. Troisi, *J. Phys. Chem. C* **122**, 18336 (2018).

(S4) C. Lubich, *From Quantum to Classical Molecular Dynamics: Reduced Models and Numerical Analysis* (European Mathematical Society Publishing House, Zuerich, Switzerland, 2008).

(S5) H.-C. Jankowiak, J. L. Stuber, R. Berger, *J. Chem. Phys.* **127**, 234101 (2007).

(S6) F. Santoro, A. Lami, R. Improta, J. Bloino, V. Barone, *J. Chem. Phys.* **128**, 224311 (2008).

(S7) R. Borrelli, A. Capobianco, A. Landi, A. Peluso, *Phys. Chem. Chem. Phys.* **17**, 30937 (2015).

(S8) R. Borrelli, A. Peluso, *J. Chem. Phys.* **129**, 064116 (2008).

(S9) F. Santoro, R. Improta, A. Lami, J. Bloino, V. Barone, *J. Chem. Phys.* **126**, 084509 (2007).

(S10) A. Capobianco, A. Landi, A. Peluso, *Phys. Chem. Chem. Phys.* **19**, 13571 (2017)

(S11) T. Chakraborty, **Charge Migration in DNA: Perspectives from Physics, Chemistry, and Biology**, Springer Berlin Heidelberg (2007).

(S12) A. Landi, R. Borrelli, A. Capobianco, A. Peluso, *J. Phys. Chem. Lett.* **10**, 1845 (2019)

(S13) F. Duschinsky, *Acta Phisicochim. URSS* **7**, 551 (1937).

(S14) A. Peluso, F. Santoro, G. Del Re, *Int. J. Quantum Chem.* **63**, 233 (1997).

(S15) E. V. Doktorov, I. A. Malkin, V. I. Manko, *J. Mol. Spec.* **56**, 1 (1975).

(S16) B. Giese, J. Amaudrut, A. Köhler, M. Spormann, S. Wessely, *Nature* **412**, 318 (2001)

(S17) S. Miertuš, E. Scrocco, J. Tomasi, *Chem. Phys.* **55**, 117 (1981).

(S18) M. J. Frisch, *et al.*, Gaussian 09 Revision D.01. Gaussian Inc. Wallingford CT 2009.

(S19) R. Borrelli, A. Peluso, MolFC: A program for Franck-Condon integrals calculation. Package available online at `http://www.theochem.unisa.it`.

(S20) R. Borrelli, A. Peluso, *J. Chem. Phys.* **119**, 8437 (2003).

(S21) R. Borrelli, A. Peluso, *J. Chem. Phys.* **125**, 194308 (2006).

(S22) A. Peluso, R. Borrelli, A. Capobianco, *J. Phys. Chem. A* **113**, 14831 (2009).

(S23) A. Capobianco, R. Borrelli, C. Noce, A. Peluso, *Theor. Chem. Acc.* **131**, 1181 (2012).

(S24) R. Borrelli, A. Capobianco, A. Peluso, *Can. J. Chem.* **91**, 495 (2013).

(S25) R. Borrelli, A. Peluso, *J. Chem. Phys.* **128**, 044303 (2008).

(S26) T. J. Park, J. C. Light, *J. Chem. Phys.* **85**, 5870 (1986).
